# The Influence of Variable Rainfall Frequency on Germination and Early Growth of Shade-Tolerant Dipterocarp Seedlings in Borneo

**DOI:** 10.1371/journal.pone.0070287

**Published:** 2013-07-24

**Authors:** Michael J. O’Brien, Christopher D. Philipson, John Tay, Andy Hector

**Affiliations:** 1 Institute of Evolutionary Biology and Environmental Studies, University of Zurich, Zurich, Switzerland; 2 School of International Tropical Forestry, University Malaysia Sabah, Kota Kinabalu, Sabah, Malaysia; Cirad, France

## Abstract

Climate change induced alterations to rainfall patterns have the potential to affect the regeneration dynamics of plant species, especially in historically everwet tropical rainforest. Differential species response to infrequent rainfall may influence seed germination and seedling establishment in turn affecting species distributions. We tested the role of watering frequency intervals (from daily to six-day watering) on the germination and the early growth of Dipterocarpaceae seedlings in Borneo. We used seeds that ranged in size from 500 to 20,000 mg in order to test the role of seed mass in mediating the effects of infrequent watering. With frequent rainfall, germination and seedling development traits bore no relationship to seed mass, but all metrics of seedling growth increased with increasing seed mass. Cumulative germination declined by 39.4% on average for all species when plants were watered at six-day intervals, and days to germination increased by 76.5% on average for all species from daily to six-day intervals. Final height and biomass declined on average in the six-day interval by 16% and 30%, respectively, but the percentage decrease in final size was greater for large-seeded species. Rooting depth per leaf area also significantly declined with seed mass indicating large-seeded species allocate relatively more biomass for leaf production. This difference in allocation provided an establishment advantage to large-seeded species when water was non-limiting but inhibited their growth under infrequent rainfall. The observed reduction in the growth of large-seeded species under infrequent rainfall would likely restrict their establishment in drier microsites associated with coarse sandy soils and ridge tops. In total, these species differences in germination and initial seedling growth indicates a possible niche axis that may help explain both current species distributions and future responses to climate change.

## Introduction

Germination and early seedling growth are highly susceptible to changes in climatic conditions such as temperature and water availability [1]–[4]. In aseasonal tropical forests, species have adapted to everwet conditions which may make these systems especially sensitive to changes in the rainfall regime [5], [6]. Previous research on the effects of drought in tropical forests has mainly focused on total water deficits during periods of no rain associated with a dry season [7]–[10]. However, in moist tropical forests, the timing and variability in rainfall may have detrimental effects on regeneration regardless of total monthly rainfall [11], [12].

In Borneo, rainfall varies greatly on daily, weekly and monthly timescales despite it being classified as a moist aseasonal climate [13]. Additionally, the forests of Borneo have evolved with El Niño Southern Oscillation (ENSO) events which are associated with drier conditions and more variable rainfall, defined as short-term rainless periods followed by extreme rainfall [13], [14]. Seedling establishment in these forests may be especially susceptible to infrequent rainfall because trees from the family Dipterocarpaceae (dipterocarps) which dominate the primary forest canopy often have recalcitrant seeds (i.e. short viability with no soil seed banks due to desiccation sensitivity), and their seedling recruitment relies on the episodic mast fruitings, which commonly coincide with ENSO events [14], [15]. Furthermore, the effect of rainfall variability on plant growth is increasingly relevant as global precipitation cycles are expected to intensify with climate change [10], [16], [17]. In the tropics, an increase in both frequency and intensity of ENSO events could have substantial impacts on tropical forest dynamics, especially at the sensitive seed and seedling stage [12], [13], [16], [18]–[21].

Seed size is an important trait which determines species success to climatic stress [1], [22]. Larger seeds often have deeper root extension beyond the drying soil profile and greater sugar reserves which provides a competitive advantage under stressful drought conditions [1], [2], [22]–[25]. For example, Daws et al. [4] demonstrated that larger-seeded species were able to germinate at lower water potentials, implying tolerance to drought-imposed desiccation. The success of seedling establishment under infrequent rainfall may be directly related to seed mass [22].

We germinated seeds under multiple different frequencies of water availability to examine the effects of rainfall variability on early seedling establishment. We used species spanning a range of more than two orders of magnitude in seed size to assess the importance of seed mass in mediating the potential negative impacts of infrequent rainfall. We examined the role of seed mass in seed germination and seedling growth by measuring both pre- and post-germination response.

## Methods

### Ethics Statement

Approval for research at this site was given by the Malaysian Economic Planning Unit (EPU Permit: #2738 UPE: 40/200/19/2640).

### Study Site

The experiment was conducted between 4 August and 8 November 2010 at the Sabah Biodiversity Experiment (SBE; N05°05′20′′ E117°38′32′′; 102 MASL). This site is located ≈22 km north of Danum Valley Field Centre (DVFC) in the state of Sabah, Malaysia [26]. Mean annual rainfall (s.e.) from 1986–2010 from DVFC was 2848.5 (94.0) mm. This experiment was conducted *ex situ* under two layers of 70% shade-cloth and thin transparent polyethylene sheeting to exclude rainfall. The shade-houses at the SBE are raised with grated flooring, excluding large mammals but not small rodents and insects. The daily mean temperature (s.e.) under the polyethylene sheeting during the course of the experiment was 25.3°C (0.08) with a minimum of 21.5°C and a maximum of 34°C. The mean (s.e.) percent direct light was 4.7% (0.1) and a red:far-red ratio of 1.11 (0.01) (measured by simultaneous shade-house and open sky photosynthetically active radiation sensors (SKP 210 quantum sensor; Skye instruments LTD, Llandrindod Wells, Powys, UK)) which created a light environment similar to a small gap in the forest understory. A nearby forest gap (approximately 8 m^2^) in the Malua Forest Reserve had 4.98% (0.002) direct light and a red:far-red ratio of 1.04 (0.002).

### Seed Collection

Fruits of eight dipterocarps were collected from the Malua Forest Reserve surrounding the SBE during the masting event which began in late July 2010. Species were selected to ensure a variety of genera, seed size and morphological characteristics (Table 1). Visibly healthy seeds (i.e. free of fungus, decay and herbivore damage) were checked for maturity by opening five to ten seeds per species and ensuring that the radicle was fully formed. Additionally, seeds were checked for health in water (i.e. seeds that sank in a basin of water were deemed healthy). Fruit wings were removed, and individual seeds without wings were weighed for seed mass. An additional 50 seeds of every species were used to generate estimates of seed dry biomass. These seeds were weighed, dried at 64°C to a constant weight and reweighed. Regressions were developed for each species relating dry and wet mass.

**Table 1:** Summary table of seed traits of dipterocarp species ordered from largest to smallest seed mass.

Species	Wet mass (mg)	Dry mass (mg)	Seed description^a^	Days to germination^a^
(Acronym)	Mean (95% CI)	Mean (95% CI)		
*Shorea macrophylla*	55229	21248	thick woody seed	14–37
Ashton (SM)	(54606–55853)	(20996–21500)	coat, recalcitrant	
*Parashorea tomentella*	6533	4228	thick woody seed	14–184
Meijer (PT)	(5910–7157)	(3976–4480)	coat, recalcitrant	
*Dryobalanops lanceolata*	6483	2714	green soft seed	7–13
Burck (DL)	(6042–6924)	(2536–2892)	coat, recalcitrant	
*Parashorea malaanonan*	3840	1760	thick woody seed	14–184
Merr. (PM)	(3217–4464)	(1508–2012)	coat, recalcitrant	
*Hopea nervosa*	2909	1272	thin hard seed	8–48
King (HN)	(2285–3533)	(1020–1523)	coat, recalcitrant	
*Shorea beccariana*	2756	944	thin hard seed	Unknown
Burck (SB)	(2133–3380)	(742–1245)	coat, recalcitrant	
*Shorea parvifolia*	853	514	thin hard seed	7–52
Dyer (SP)	(230–1477)	(262–766)	coat, recalcitrant	
*Shorea argentifolia*	809	485	thin hard seed	8–15
Symington (SA)	(230–1477)	(233–737)	coat, recalcitrant	

a Descriptions and germination range taken from [30] and recalcitrance taken from KEW database.

### Watering Frequency and Planting

**Figure 1:**
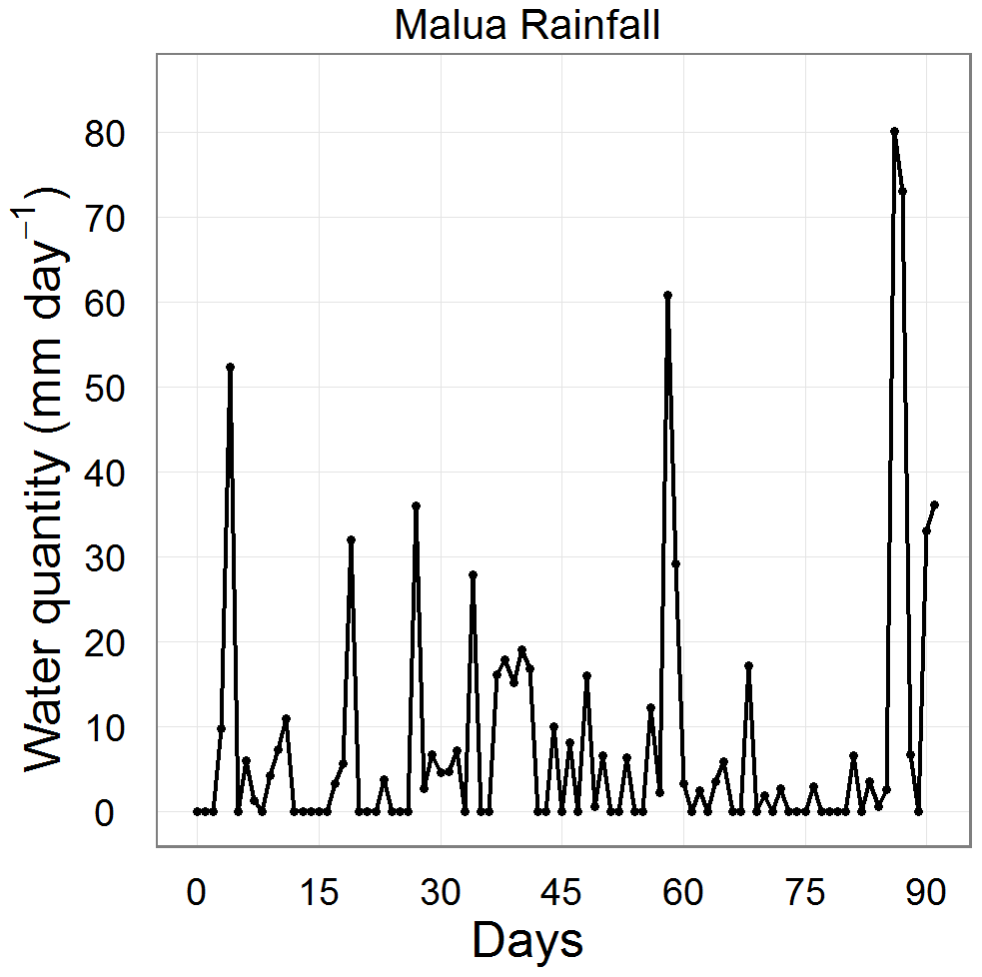
Local rainfall at Malua forest during the experiment. Daily rainfall over the period of the experiment. Total rainfall was approximately 724 mm over the course of 90 days with 44 near rainless days (<1 mm day^−1^).

**Figure 2:**
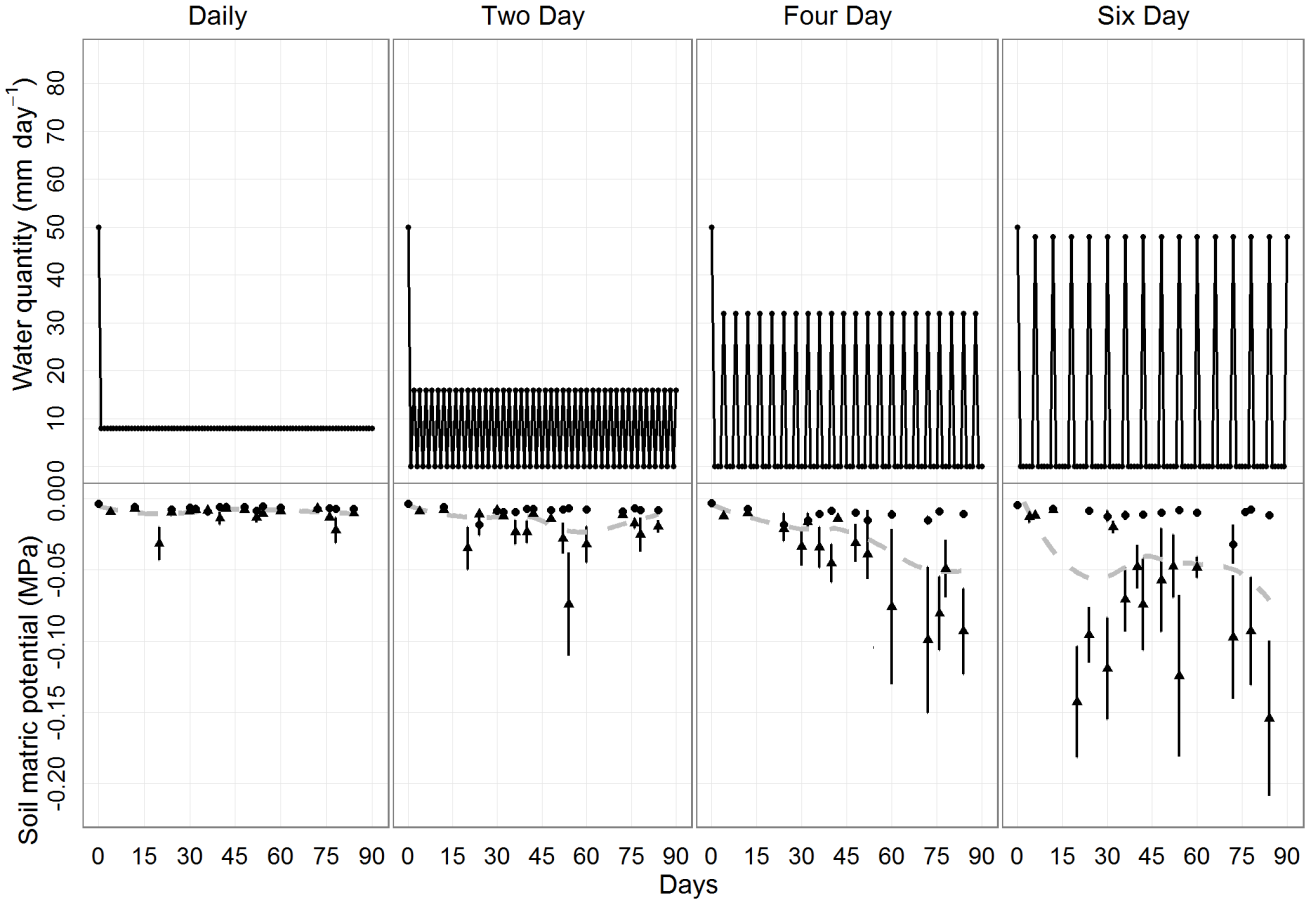
The effect of watering pattern on soil matric potential. The frequency of watering per treatment as a function of time. Each treatment received 720 mm of water. The lower panels show the change in soil matric potential with each treatment (pre- and post-watering measures are jittered for readability). The grey line represents a loess curve of change in the smoothed average through time.

Pots were filled with homogenized forest soil obtained from the Innoprise-FACE Foundation Rainforest Rehabilitation Project (http://www.face-thefuture.com) (pot sizes: 7.0×23.0 cm for large-seeded *S. macrophylla* to allow greater soil volume; 4.5×22.0 cm for all other species). The soil used was classified as clay comprised of 50% clay, 30% silt and 20% sand similar to upslope sites of the Malua Forest Reserve. Total monthly rainfall (30 day period) was set at 240 mm and dispensed at four frequencies to assess the role of rainfall pattern on germination and seedling development. The water quantity was calculated based on the area of the pots and the millimeters of water per day for each watering frequency. This quantity equated to approximately 13, 25, 51 and 63 ml pot^−1^ per watering for daily, two, four and six-day frequencies. This watering regime altered frequency while sustaining an equal monthly rainfall, and provided seedlings with similar total water as natural rainfall during that period (Figure 1). Watering frequencies were chosen based on analysis of monthly rainfall records at the DVFC. The daily frequency was based on the maximum number of days with rain in a 30 day period and six-day frequency was based on the minimum number of days with rain in a 30 day period from historical rainfall records (5 days in April 1998; [13]). The two and four-day frequencies provided approximately the mean and 25% quantile of rainfall days. Furthermore, soil matric potentials during the course of the experiment remained within the range of natural values recorded in the forest at DVFC (Figure 2; [5]).

One hundred seeds of each species were placed in pots and randomly assigned to one of the four treatments (25 seeds per species per treatment). Seeds were placed on their side on the soil surface to replicate natural seed position in the forest and allow for monitoring of germination. All pots were watered to saturation prior to planting (Figure 2).

### Soil Matric Potential

Pots were measured for volumetric soil water content with an ML2x Theta Probe and HH2 moisture meter (Delta-T Devices, Burwell, Cambridge, UK) from day zero to eighty-four. Measurements were taken before and one hour after watering to determine minimum water content and the extent to which the soil was rehydrated. To calculate a drying curve for the clay soil used in the experiment, the relationship between soil matric potential and volumetric soil moisture was determined using the filter paper method [27]. A range of volumetric soil moisture from 1.5% to 46% was used to develop two linear equations between volumetric soil moisture and soil matric potential. Two equations were required because soil matric potential declined at a faster rate below 28% volumetric soil moisture (Figure S1).

### Seed and Seedling Monitoring

Seeds were monitored daily for germination (radicle emergence) and mortality. Only seeds with visible signs of mortality were recorded (i.e. fungus, mammal browse, insect browse, or desiccation). Following germination, seedlings were monitored daily for mortality and leaf formation. All surviving seedlings were harvested on their 60^th^ day. Roots were extracted and measured for length. Leaf photographs were taken for calculation of leaf area. Seedlings were dried to a constant temperature at 64°C, and leaves, stem and roots weighed.

### Analysis

Soil matric potential was analyzed as a function of watering frequency, pre- and post-watering measurements, species, day and their interaction with a general least squares (gls; allowing for non-constant variance of different species using the varIdent function) in the nlme package for R version 2.13.2 [28], [29]. Including a factor for species identity never improved the model fit, so it was dropped from the soil matric potential analysis.

Phenological and morphological variables (cumulative germination; number of days to germination; number of days to leaf formation; diameter (mm); height (mm); root length (mm); total dry biomass (g); leaf area (cm^2^)) in the daily watering were compared using linear mixed effects models (with species identity as a random effect) to assess differences in baseline performance across the seed mass spectrum. Seed mass was log_10_ transformed and a weighted variance for each species using the varIdent function was included for all analysis in order to meet assumptions of linearity. We analyzed the data both with and without *S. macrophylla* because a large number of the seeds of this species experienced mortality due to fungal infection. We chose to retain it in all analysis, as removing it did not qualitatively alter the results (see Figure S4, S5, S6, S7, S8, S9 without *S. macrophylla*).

The effect of water frequency on seedling variables was assessed with pooled means of all species in each watering frequency. We accounted for species variance around the pooled mean with a random effect of species varying within treatment. The lmer function in the lme4 package was used for this analysis due to the added complexity of the random effects.

In order to examine the tolerance to infrequent watering, the mean relative difference between daily and six-day watering of each species was calculated for cumulative germination, days to germination, final total biomass and height (eq. 1)
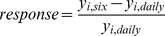
(1)Where *y_i,six_* is the mean performance of variable *i* in the six-day watering frequency, and *y_i,daily_* is the mean performance of variable *i* in the daily frequency. Relative response equates the effect of infrequent watering for a given variable in proportion to its baseline performance [12]. Large differences from zero can be interpreted as a variable which was sensitive to infrequent watering where small differences would be a variable tolerant to infrequent watering. Generalized least square models compared these proportions with species seed mass to assess the relationship between seed mass and relative response to infrequent watering for germinating seeds and early seedling growth. We examined the change in biomass allocation metrics (i.e. shoot:root (g g^−1^; SRR), root mass:total mass (g g^−1^; RMR) and root depth:leaf area (mm cm^−2^; RLA) ratios) with seed mass to explain trends in the relative response to infrequent watering using the same linear methods for assessing baseline variable differences.

## Results

### Soil Matric Potential

Pre-watering soil matric potential was significantly lower at the end of each four and six-day watering frequency but recovered to that of the daily and two-day treatments after watering (Figure 2; Table S1 [significant pre-watering*treatment interaction]). The difference between pre- and post-watering soil matric potential increased throughout the course of the experiment and at different rates for each treatment (Figure 2; Table S1 [significant three-way interaction]). Initial mean pre-watering soil matric potential for all watering frequencies was −0.004 (±0.001 s.e.) MPa (Figure 2). At the final measure on the 84^th^ day, mean pre-watering soil matric potential for daily, two, four and six-day watering was −0.01 (±0.007 s.e.), −0.02 (±0.005 s.e.), −0.09 (±0.03 s.e.) and −0.15 (±0.05 s.e.) MPa, respectively (Figure 2).

### Baseline Performance

**Figure 3:**
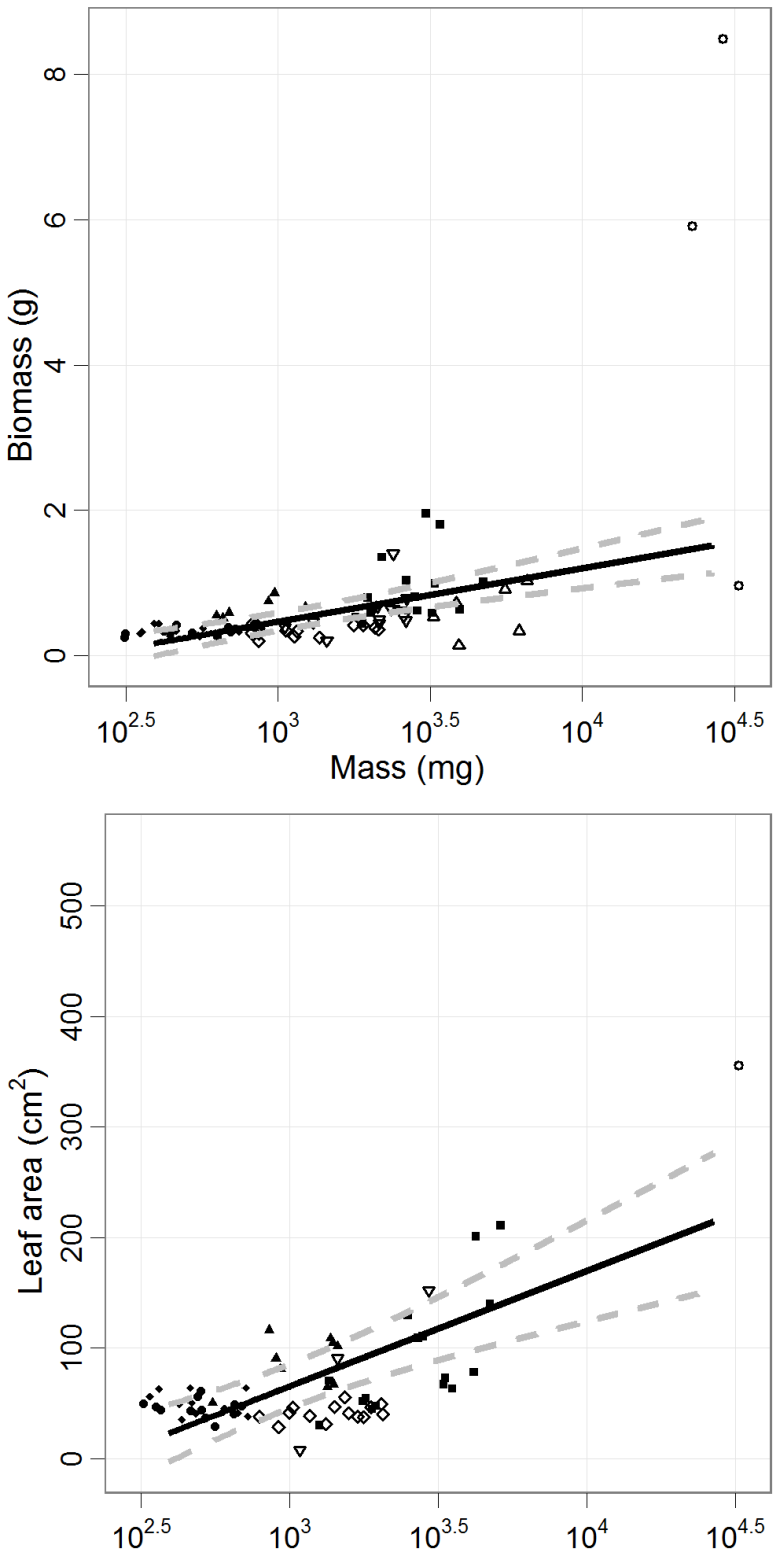
Relationship between growth and seed mass in daily watering. The relationship of baseline biomass and leaf area as a function of seed mass in the daily watering treatment (mean ±95% CI). Unequal variance was accounted for using a weighted variance for each species. Leaf area was based on the last harvest after 60 days. Points represent individual seedlings. (SM: open circle, PT: upward triangle, DL: closed square, PM: downward triangle, HN: open diamond, SB: closed triangle, SP: closed diamond, SA: closed circle).

We found species-specific differences in phenological and morphological variables (Figure S2) with growth clearly related to seed size. However, germination variables did not correlate with seed mass (Figure S2). Height, diameter, root depth, biomass and leaf area increased with seed mass by 73.5 mm (95% CI: 15.3–131.7), 1.9 mm (95% CI: 1.3–2.5), 25.0 mm (95% CI: 3.5–46.5), 0.74 g (95% CI: 0.47–1.00) and 104.4 cm^2^ (95% CI: 62.5–146.4) per log_10_ mg, respectively (Figure 3; Figure S2).

### Water Frequency, Species Characteristics and Seed Mass

**Figure 4:**
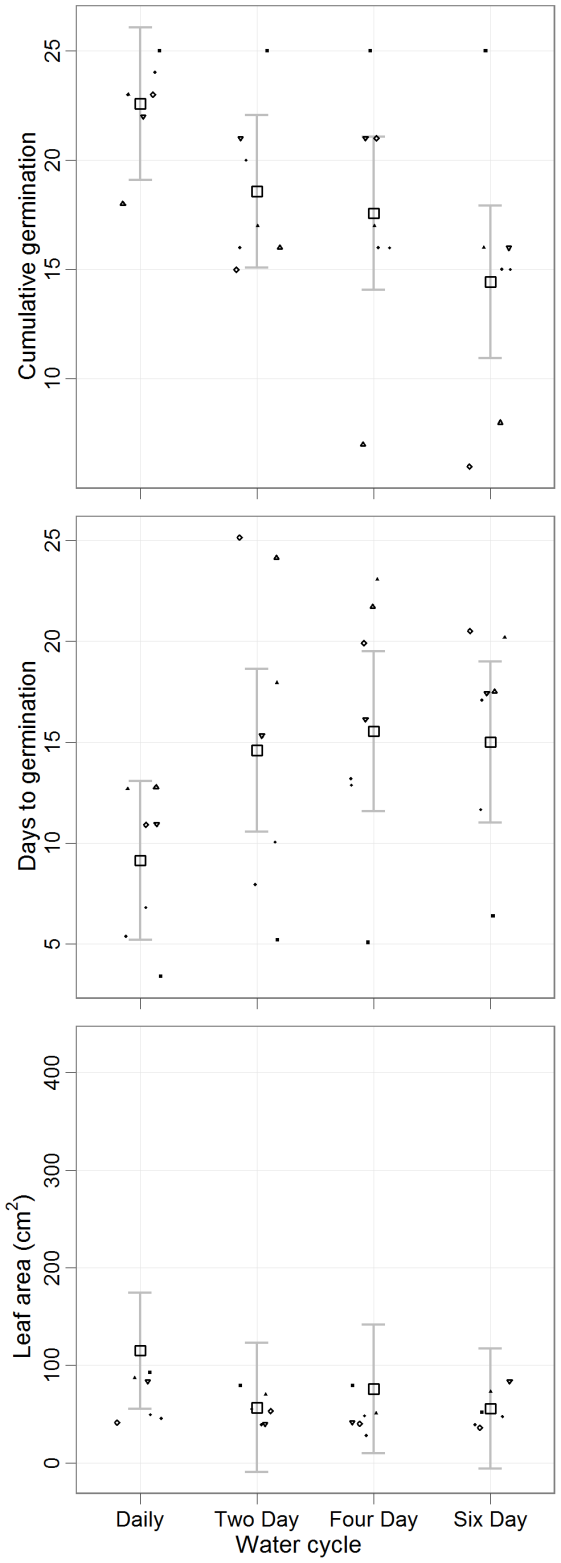
Effect of watering frequency on germination and growth. Cumulative germination, days to germination and final seedling leaf area calculated in each treatment. Variation in species and treatments was accounted for with a random effect for species in treatment. Leaf area was calculated from the last harvest after 60 days. The large open squares represent the pooled mean (±95% CI) of the eight Bornean climax species. All three traits were negatively affected by infrequent watering. Grey points represent species means for each variable in each treatment. The points are jittered for readability. (SM: open circle, PT: upward triangle, DL: closed square, PM: downward triangle, HN: open diamond, SB: closed triangle, SP: closed diamond, SA: closed circle).

Altering water frequency from daily to six-day watering negatively affected most variables. Mean (daily – six-day) cumulative germination (23–14 seeds), height (158.4–118.4 mm), diameter (3.1–1.9 mm), root depth (93.7–83.1 mm), biomass (1.1–0.4 g) and leaf area (115.0–55.6 cm^2^) declined with infrequent watering while days to germination (9–15 days) increased from daily to six-day watering, respectively (Figure 4; Figure S3). Timing of leaf formation and root depth per leaf area were not affected by infrequent watering (Figure S3).

**Figure 5:**
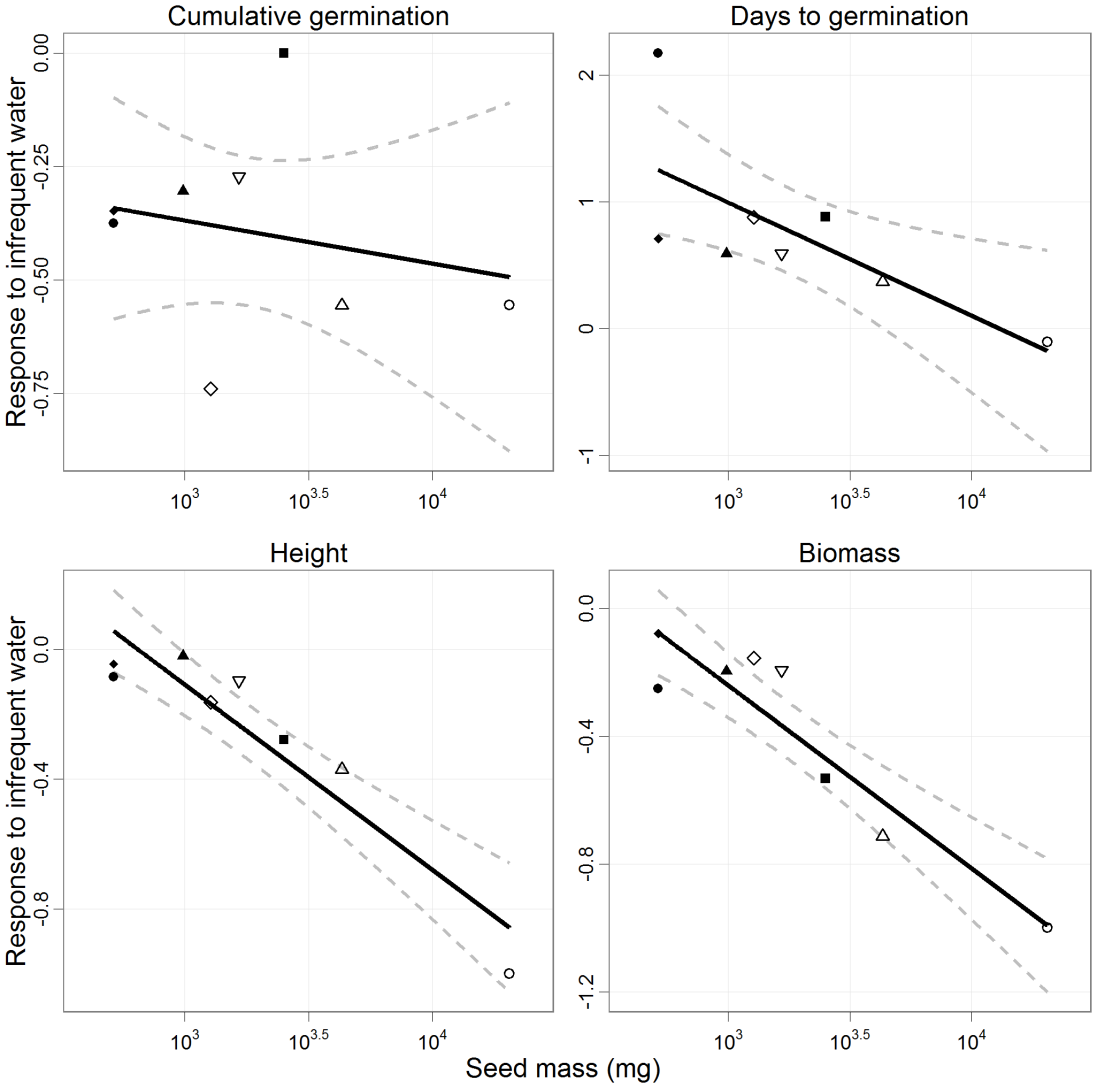
Relative response to infrequent water as a function of seed mass. Response to infrequent watering (i.e. the relative difference between daily and six-day watering) as a function of seed mass for cumulative germination, days to germination and seedling height and biomass (mean ±95% CI). No relationship existed between cumulative germination and seed mass. Germination of large seeds was more resistant to infrequent watering, but after germination large-seeded species had significantly greater declines in growth due to infrequent watering. Points represent mean values for each species. (SM: open circle, PT: upward triangle, DL: closed square, PM: downward triangle, HN: open diamond, SB: closed triangle, SP: closed diamond, SA: closed circle).

**Figure 6:**
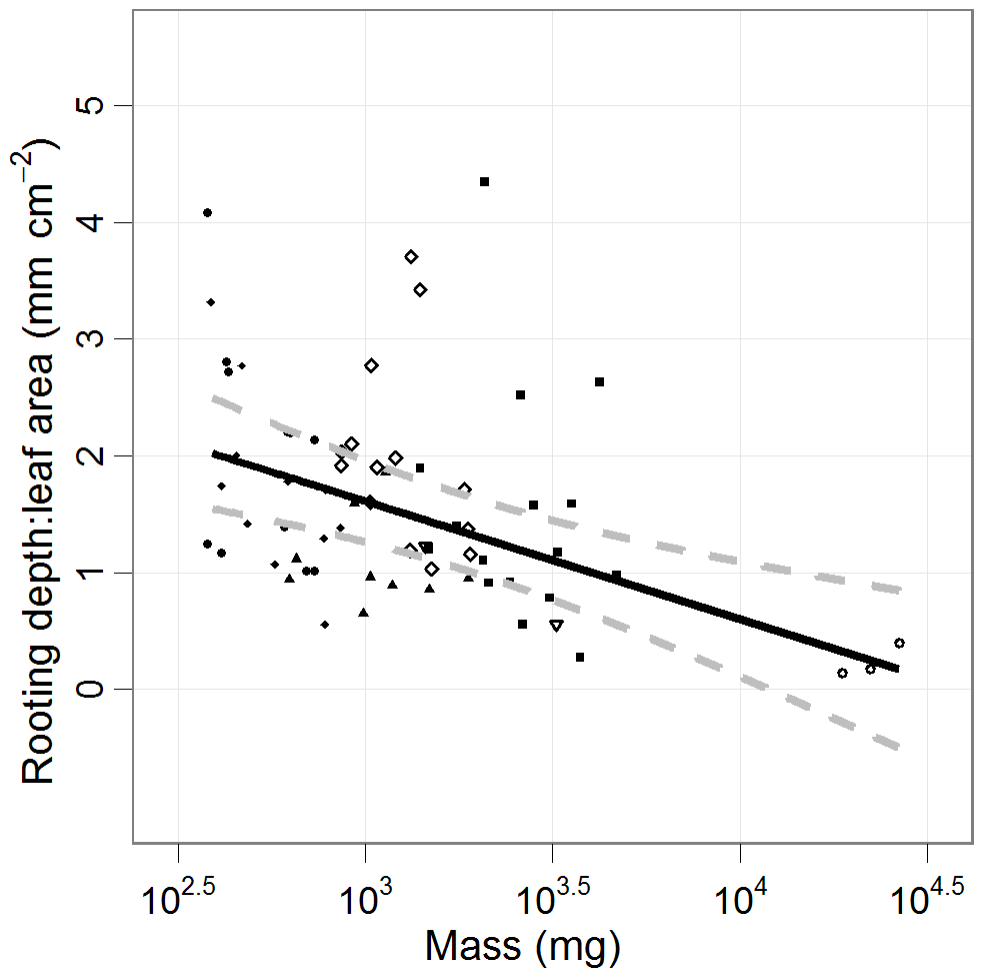
Rooting depth per leaf area as a function of seed mass. Root depth per leaf area (mean ±95% CI) as a function of seed mass. Unequal variance was accounted for using a weighted variance for each species. Large-seeded species produce much larger leaves increasing transpiration and water demand without proportionally larger rooting depth, leaving them susceptible to breaks in hydraulic conductivity and water stress. Points represent individual seedlings. (SM: open circle, PT: upward triangle, DL: closed square, PM: downward triangle, HN: open diamond, SB: closed triangle, SP: closed diamond, SA: closed circle).

There was no relationship between stress response and seed mass for cumulative germination (Figure 5). However, seed mass was related to response to infrequent water for days to germination, height, and total biomass (Figure 5). Days to germination increased by as much as 217% for *S. argentifolia* (the smallest seeded species) and as little as a 10.4% decrease for *S. macrophylla* (i.e. no effect for the largest seeded species) from daily to six-day frequency. On average a 76.5% (±23.1 s.e.) increase in days to germination was observed. The increase in days to germination declined with seed mass (slope = −0.89, 95% CI: −1.7– −0.04). Height decreased on average by 25.7% (±11.4 s.e.) for all species. Height decreased by as much as 36.9% for *P. tomentella* (for the largest seeded species surviving to 60 days) and 8.5% for *S. argentifolia* (the smallest seeded species) from daily to six-day frequency. Differences in height between treatments increased with seed mass (slope = −0.58, 95% CI: −0.79– −0.36). Biomass decreased by as much as 71.4% for *P. tomentella* and 7.9% for *S. parvifolia*, and on average decreased by 39.1% (±11.5 s.e.) for all species. Large-seeded species had greater differences in biomass between treatments (slope = −0.55, 95% CI: −0.78– −0.34). Not one seedling from the largest seeded species, *S. macrophylla*, survived to the end of the experiment in the six-day watering cycle. RLA declined with seed mass by −1.0 (95% CI: −1.53– −0.48) per log_10_ mg, but none of the alternative allocation measures showed a significant trend with seed mass (Figure 6; Figure S2).

## Discussion

This study examined the role of infrequent rainfall on seedling establishment of Bornean dipterocarp species focusing on seed mass as a mediating trait. We found that infrequent watering altered the temporal distribution of seeds and size distribution of plants across a seed mass spectrum, with infrequent rainfall favoring large-seeded species before germination but favoring small-seeded species after germination. Under the high rainfall frequency characterizing the aseasonal tropics, all seed sizes took a similar number of days to germinate, and large-seeded species grew to a larger size than small-seeded species. However, infrequent rainfall disproportionately hindered large-seeded species growth compared with that of small-seeded species, which equalized final seedling size between large and small seeded species.

### Germination

Days to germination showed no simple trends with seed mass suggesting other unmeasured seed traits were likely influential in germination. Seed coat thickness and toughness were two unmeasured traits that likely affected germination. The five species with the longest days to germination regardless of seed mass had the thickest and most lignified seed coats ([30]; Table 1; Figure 3). Alternatively, the species with the fewest days to germination (*D. lanceolata*) was the third largest seed but had a soft, green coat. Thick lignified seed coats would require a softening or breaking for the radicle to emerge. This would result from abiotic forces (e.g. the wetting and drying of the seed coat from water or diel temperature fluctuation) or biotic traits (e.g. increased emergence force from larger radicles associated with larger seeds) [25], [31], [32]. Species may also vary in response to environmental factors such as light quantity and quality and temperature that may account for unexplained variation in days to germination [3]. Therefore, the timing of germination is driven by complex interactions between seed traits and microsite conditions, which did not simply correlate with seed mass for dipterocarps [3], [31].

Infrequent rainfall inhibited days to germination for small-seeded species while large-seeded species remained unaffected (Figure 5). This temporal germination advantage favors large seeds as they reached greater height and rooting depths which could potentially inhibit establishment and growth of small-seeded species [22]. This advantage for large seeds would be more pronounced under two scenarios. First, under canopy gaps, lower humidity and higher daytime temperatures would increase seed and soil surface drying associated with infrequent rainfall, possibly slowing small seed germination to a greater extent [2], [3], [8], [12], [32]. Second, current climate projections suggest that rainfall variability will increase. Walsh and Newbery [13] have shown that consecutive months with short-term drought have been more frequent since the last decades of the 20^th^ century. For example, from September 1997 to May 1998 there were four rainless periods of at least 10 days [13]. More recently, from February 2010 to April 2010 there were three rainless periods of at least 7 days (O’Brien, unpublished data). Under more frequent or longer rainless periods, large-seeded species would have a germination advantage [13], [20]. However, dipterocarp seeds likely have a threshold to infrequent rainfall that, if reached during extreme ENSO events, may cause total regeneration failure, especially since many dipterocarps are desiccation sensitive [14], [30].

Infrequent rainfall reduced cumulative germination, but the reduction was independent of seed mass (Figure 5). Natural rainfall patterns during the course of this experiment showed days of high rain and more than ten periods with consecutive no rain days, six of which were >2 days long (Figure 2). Overall seed germination in the six-day watering was reduced by 37% relative to the daily watering, which would have marginal impacts on seedling establishment under such ubiquitous seed production. However, greater declines should be expected under projected climate scenarios where extended dry periods (>10 days) are becoming more frequent [12], [13].

### Seedling Growth

Large-seeded species had greater growth under frequent rainfall (Figure 3). The greater stored non-structural carbohydrates often associated with larger seeds allows increased biomass growth and greater absolute height growth before leaf development [1], [24], [33]. Although absolute growth was greater for large-seeded species after 60 days, small-seeded species commonly have higher relative growth rate and may overtake large-seeded species given longer time periods [34], [35].

All growth metrics showed a decline with infrequent watering. The magnitude of the effect was dependent on the metric with total biomass and leaf area having the greatest decline and root depth having the smallest. The overall decline in growth was likely due to either water limitation which inhibited cell expansion and division or due to the rapid shift from an aerobic to anaerobic environment [36]. Daily and two-day watering maintained soil matric potential at a constant level throughout the experiment while four and six-day watering caused levels more than ten times lower than daily watering. Soil drying also increased throughout the course of the experiment (Figure 2) because seedlings began establishing and taking up water. As root systems depleted soil water to meet growth and transpiration demands, drought stress increased, and growth was limited. However, soil matric potentials never dropped much below −0.2 MPa which is a relatively mild drought stress, and we hypothesize that drying would be more significant in the forest setting. Regardless of the extent of drought stress experienced by the seedlings, soil drying followed by rapid water saturation may alone be enough of a stress to cause internal water deficits [37], [38].

Growth of large-seeded species was more inhibited by infrequent watering than growth of small-seeded species (Figure 5). After 60 days of growth, a difference of only 24 mm between *D. lanceolata* (a large seeded species) and *S. argentifolia* (the smallest seeded species) was observed under six-day watering versus 76 mm under daily watering. If this trend were to continue with longer rainless periods, it would alter the competitive rank of the species. The effect was even more pronounced for biomass in which the large-seeded *P. tomentella* (0.61 g –0.18 g) declined to below the mass of the smallest seeded *S. argentifolia* (0.34 g –0.25 g). Although soil matric potential did not show a significant difference between species, seedlings with greater water uptake could deplete soil water from their immediate soil rhizosphere causing a break in water conductivity and a more intense drought effect [39]. Additionally, the drying and wetting of the soil in the infrequent watering treatment, which would alter nutrient availability and soil texture, could have negative effects on seedling growth regardless of deficit [37], [38]. We hypothesize that small-seeded species are less susceptible to these fluctuations in soil properties because of their finer roots relative to large-seeded species, which allows them to more easily alter root growth in the varying soil environment. However, research focusing directly on seedling root plasticity with a fluctuating water table is necessary to elucidate the mechanism driving this process.

The inverse relationship between rooting depth per leaf area and seed mass (Figure 6) may explain the increased effect on large-seeded species. In support of this result, Engelbrecht et al. [12] also found significantly larger rooting depth per leaf area of smaller seeded species. Greater leaf area per unit of root depth increases the risk of desiccation as high transpiration demands are not met leading to a drawdown of the soil water [39]. For a seedling to sustain the required hydraulic conductivity, it must access an increased soil volume [39]. Large-seeded species produced greater leaf area which equated to increased transpiration and subsequent water loss. At the early establishment phase, large seedlings would be at a disadvantage under infrequent rainfall especially in rapidly drying sites such as coarse textured soils and large canopy gaps [2], [8], [32], [39]. We recognize that seed mass is a simplification of multiple traits into a single dimension, and physiological differences in water use efficiency or carbohydrate storage may provide further explanations for the sensitivity of species [40].

Our results have implications for species distributions in Bornean forests. Large-seeded species may preferentially establish in wetter areas which sustain consistent soil moisture such as lowland riparian zones, and small-seeded species may persist in sites with well-drained soils which have a more fluctuating water table. Recent work done in Lambir Hills National Park, Sarawak, Malaysia found an increase in mean seed mass along a soil moisture and fertility gradient with the largest seed masses found on fine clayey soil textures [41], which supports our seed size-sensitivity relationship.

### Conclusions

This study demonstrates differential responses of large- and small-seeded species to watering frequency and temporal distribution of rainless periods. The timing of rainless periods (before or after germination) altered the competitive advantage between seed sizes. Short-term rainless periods delayed germination of small-seeded species which gave a temporal advantage to large-seeded species, but at the seedling establishment phase, large seedlings resulting from larger seeds were susceptible to fluctuations in water which inhibited growth. Current climate change projections of reduced and more variable rainfall could further alter species germination and establishment patterns which would have long-term effects on community composition and species distributions.

## Supporting Information

Figure S1 **The curves used to estimate matric potential from volumetric moisture.** The matric potential declines at a much faster rate below approximately 28% volumetric moisture (5.477–0.1591×volumetric; R^2^ = 0.977) than above (2.307–0.044 × volumetric; R^2^ = 0.981).
(TIF)

Figure S2 **Baseline variables in of seeds and seedlings in daily watering.** The relationship of baseline phenological and morphological characteristics with seed mass for germinating seeds and seedlings in the daily watering treatment. Seedling growth and allocation variables were based on the last harvest after 60 days. Solid lines represent model predictions with 95% CIs. Points represent individual observations (SM: open circle, PT: upward triangle, DL: closed square, PM: downward triangle, HN: open diamond, SB: closed triangle, SP: closed diamond, SA: closed circle). Log transforming growth supported untransformed results and was therefore not used for the analysis.
(TIF)

Figure S3 **Effect of watering frequency on seed and seedling variables.** The effect of water frequency on seed and seedling variables for seven Bornean shade-tolerant species pooled. Seedling variables were based on the last harvest after 60 days. Most variables were negatively affected by infrequent watering. Individual species points were jittered for readability. Open squares represent model predictions with 95% CIs. The smaller points represent mean for each species in each treatment. (SM: open circle, PT: upward triangle, DL: closed square, PM: downward triangle, HN: open diamond, SB: closed triangle, SP: closed diamond, SA: closed circle)
(TIF)

Figure S4 **Baseline variables in of seeds and seedlings in daily watering without SM.** The relationship of baseline phenological and morphological characteristics with seed mass for germinating seeds and seedlings in the daily watering treatment. Seedling growth and allocation variables were based on the last harvest after 60 days. Solid lines represent model predictions with 95% CIs. Points represent individual observations (PT: upward triangle, DL: closed square, PM: downward triangle, HN: open diamond, SB: closed triangle, SP: closed diamond, SA: closed circle). Log transforming growth supported untransformed results and was therefore not used for the analysis.
(TIF)

Figure S5 **Effect of watering frequency on seed and seedling variables without SM.** The effect of water frequency on seed and seedling variables for seven Bornean shade-tolerant species pooled. Seedling variables were based on the last harvest after 60 days. Most variables were negatively affected by infrequent watering. Individual species points were jittered for readability. Open squares represent model predictions with 95% CIs. The smaller points represent mean for each species in each treatment. (PT: upward triangle, DL: closed square, PM: downward triangle, HN: open diamond, SB: closed triangle, SP: closed diamond, SA: closed circle)
(TIF)

Figure S6 **Relationship between growth and seed mass in daily watering without SM.** The relationship of biomass and leaf area as a function of seed mass in the daily watering treatment (mean ±95% CI). Unequal variance was accounted for using a weighted variance for each species. Leaf area was based on the last harvest after 60 days. Points represent individual seedlings. (PT: upward triangle, DL: closed square, PM: downward triangle, HN: open diamond, SB: closed triangle, SP: closed diamond, SA: closed circle)
(TIF)

Figure S7 **Effect of watering frequency on germination and growth without SM.** Cumulative germination, days to germination and final seedling leaf area calculated in each treatment. Variation in species and treatments was accounted for with a random effect for species in treatment. Leaf area was calculated from the last harvest after 60 days. The large open squares represent the pooled mean (±95% CI) of the eight Bornean climax species. All three traits were negatively affected by infrequent watering. Grey points represent species means for each variable in each treatment. The points are jittered for readability. (PT: upward triangle, DL: closed square, PM: downward triangle, HN: open diamond, SB: closed triangle, SP: closed diamond, SA: closed circle)
(TIF)

Figure S8 **Relative response to infrequent water as a function of seed mass without SM.** Response to infrequent watering (i.e. the relative difference between daily and six-day watering) as a function of seed mass for cumulative germination, days to germination and seedling height and biomass (mean ±95% CI). No relationship existed between cumulative germination and seed mass. Germination of large seeds was more resistant to infrequent watering, but after germination large-seeded species had significantly greater declines in growth due to infrequent watering. Points represent mean values for each species. (PT: upward triangle, DL: closed square, PM: downward triangle, HN: open diamond, SB: closed triangle, SP: closed diamond, SA: closed circle)
(TIF)

Figure S9 **Rooting depth per leaf area as a function of seed mass without SM.** Root depth per leaf area (mean ±95% CI) as a function of seed mass. ). Unequal variance was accounted for using a weighted variance for each species. Large-seeded species produce much larger leaves increasing transpiration and water demand without proportionally larger rooting depth, leaving them susceptible to breaks in hydraulic conductivity and water stress. Points represent individual seedlings. (PT: upward triangle, DL: closed square, PM: downward triangle, HN: open diamond, SB: closed triangle, SP: closed diamond, SA: closed circle)
(TIF)

Table S1 **Summary of significance for parameters explaining soil matric potential.** Species was removed from the model as it was never significant nor did it improve the fit of the model.
(DOCX)
