# Supplementary figures and images for: The Influence of Variable Rainfall Frequency on Germination and Early Growth of Shade-Tolerant Dipterocarp Seedlings in Borneo
Source: PLoS One. 2013 Jul 24;8(7):e70287. doi: 10.1371/journal.pone.0070287 (PMC3722165; doi:10.1371/journal.pone.0070287)

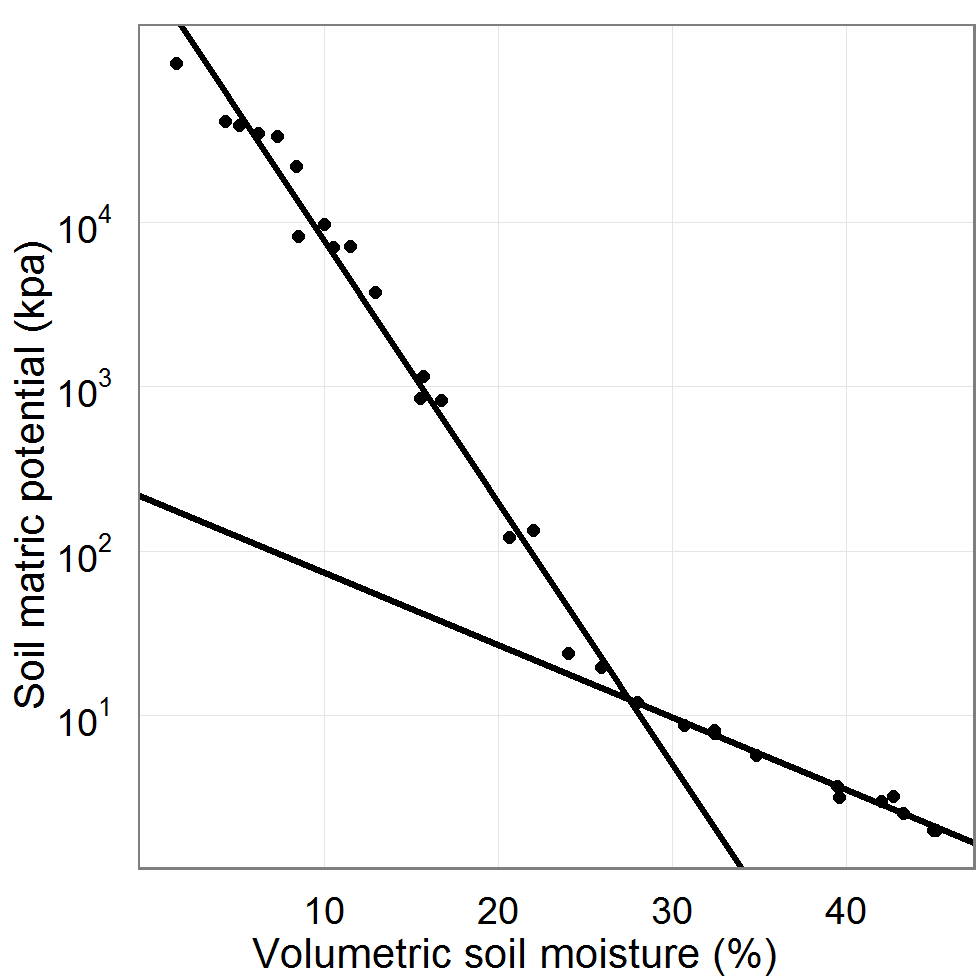

Supplement: Figure S1 — The curves used to estimate matric potential from volumetric moisture. The matric potential declines at a much faster rate below approximately 28% volumetric moisture (5.477–0.1591×volumetric; R2 = 0.977) than above (2.307–0.044 × volumetric; R2 = 0.981). (TIF) [file pone.0070287.s001.tif]

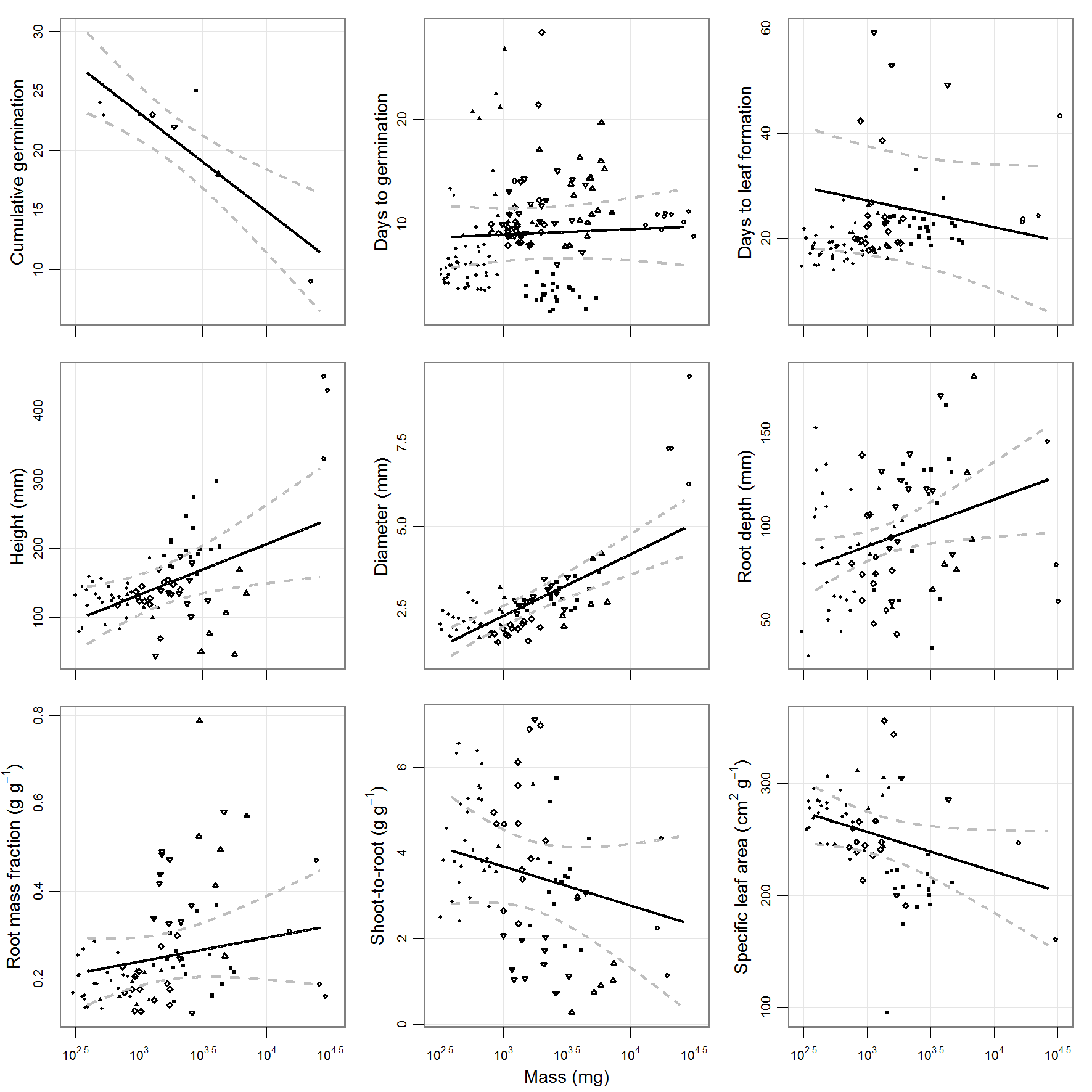

Supplement: Figure S2 — Baseline variables in of seeds and seedlings in daily watering. The relationship of baseline phenological and morphological characteristics with seed mass for germinating seeds and seedlings in the daily watering treatment. Seedling growth and allocation variables were based on the last harvest after 60 days. Solid lines represent model predictions with 95% CIs. Points represent individual observations (SM: open circle, PT: upward triangle, DL: closed square, PM: downward triangle, HN: open diamond, SB: closed triangle, SP: closed diamond, SA: closed circle). Log transforming growth supported untransformed results and was therefore not used for the analysis. (TIF) [file pone.0070287.s002.tif]

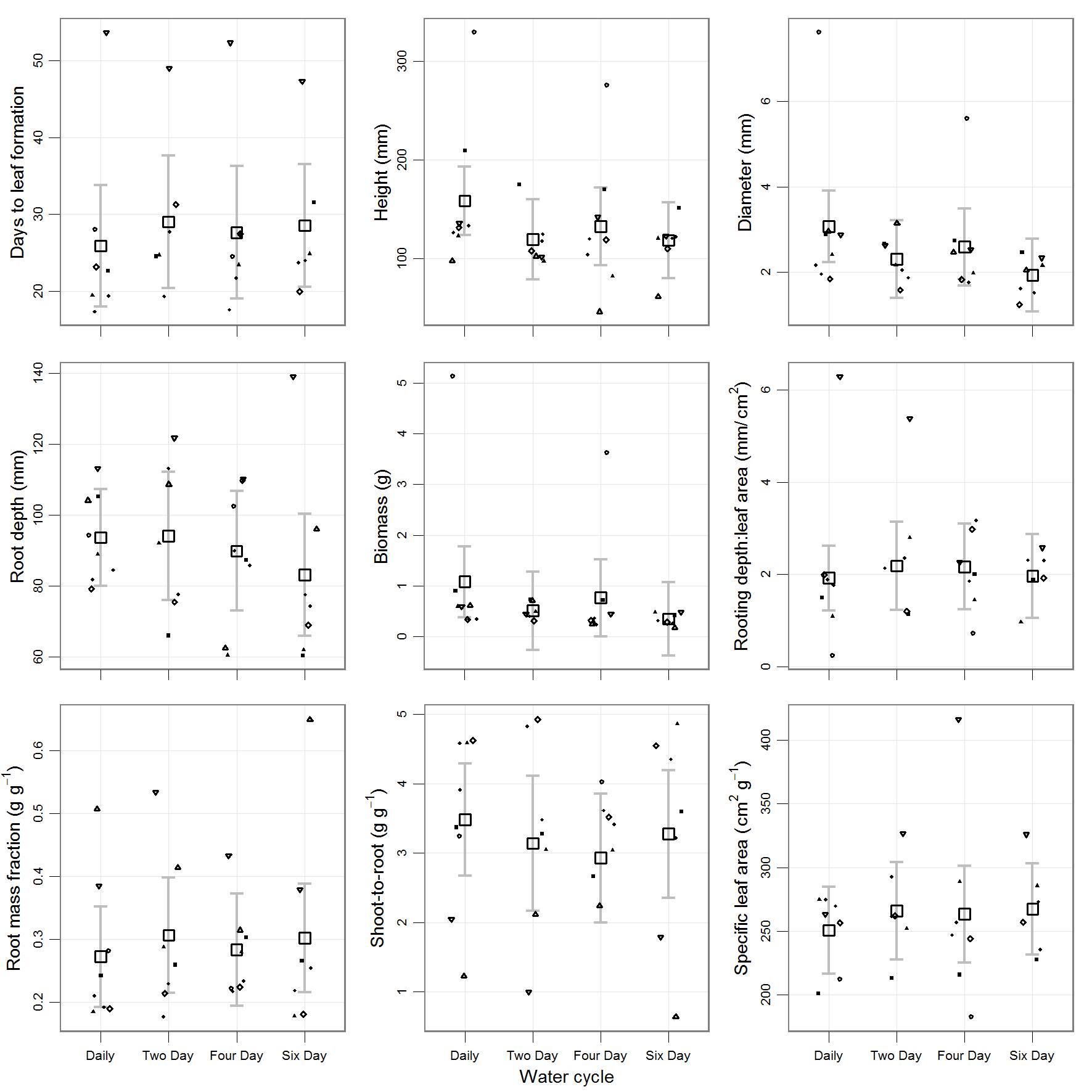

Supplement: Figure S3 — Effect of watering frequency on seed and seedling variables. The effect of water frequency on seed and seedling variables for seven Bornean shade-tolerant species pooled. Seedling variables were based on the last harvest after 60 days. Most variables were negatively affected by infrequent watering. Individual species points were jittered for readability. Open squares represent model predictions with 95% CIs. The smaller points represent mean for each species in each treatment. (SM: open circle, PT: upward triangle, DL: closed square, PM: downward triangle, HN: open diamond, SB: closed triangle, SP: closed diamond, SA: closed circle) (TIF) [file pone.0070287.s003.tif]

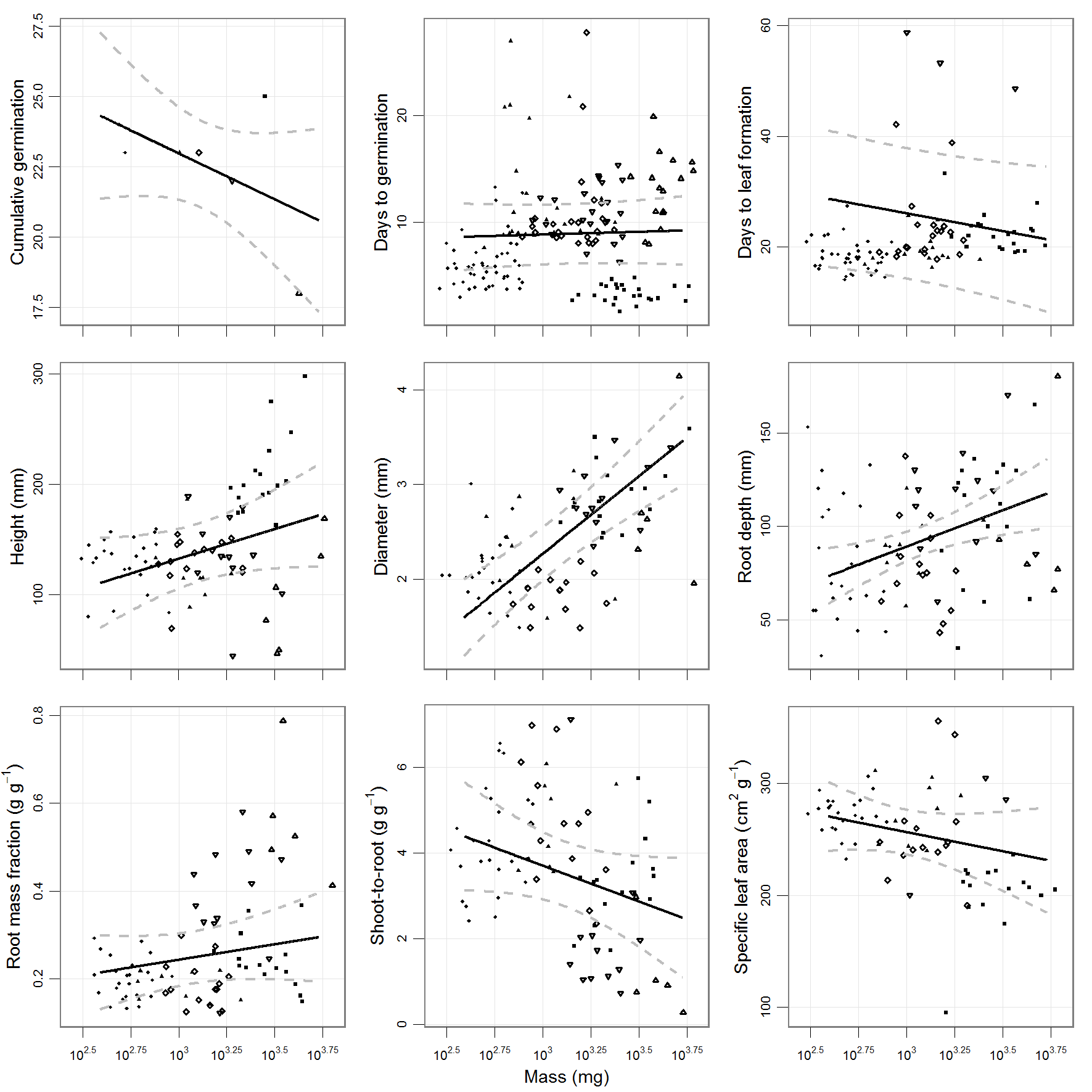

Supplement: Figure S4 — Baseline variables in of seeds and seedlings in daily watering without SM. The relationship of baseline phenological and morphological characteristics with seed mass for germinating seeds and seedlings in the daily watering treatment. Seedling growth and allocation variables were based on the last harvest after 60 days. Solid lines represent model predictions with 95% CIs. Points represent individual observations (PT: upward triangle, DL: closed square, PM: downward triangle, HN: open diamond, SB: closed triangle, SP: closed diamond, SA: closed circle). Log transforming growth supported untransformed results and was therefore not used for the analysis. (TIF) [file pone.0070287.s004.tif]

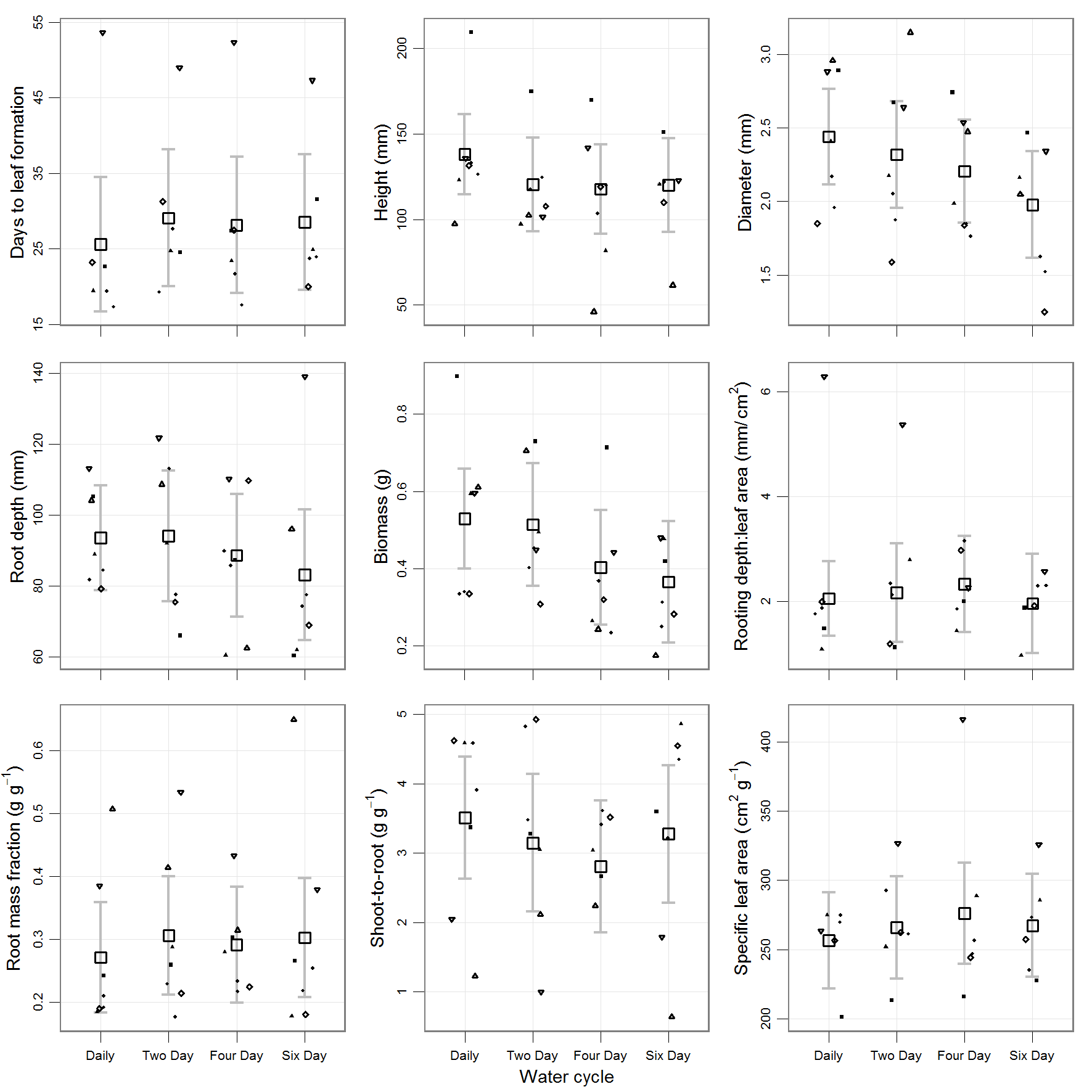

Supplement: Figure S5 — Effect of watering frequency on seed and seedling variables without SM. The effect of water frequency on seed and seedling variables for seven Bornean shade-tolerant species pooled. Seedling variables were based on the last harvest after 60 days. Most variables were negatively affected by infrequent watering. Individual species points were jittered for readability. Open squares represent model predictions with 95% CIs. The smaller points represent mean for each species in each treatment. (PT: upward triangle, DL: closed square, PM: downward triangle, HN: open diamond, SB: closed triangle, SP: closed diamond, SA: closed circle) (TIF) [file pone.0070287.s005.tif]

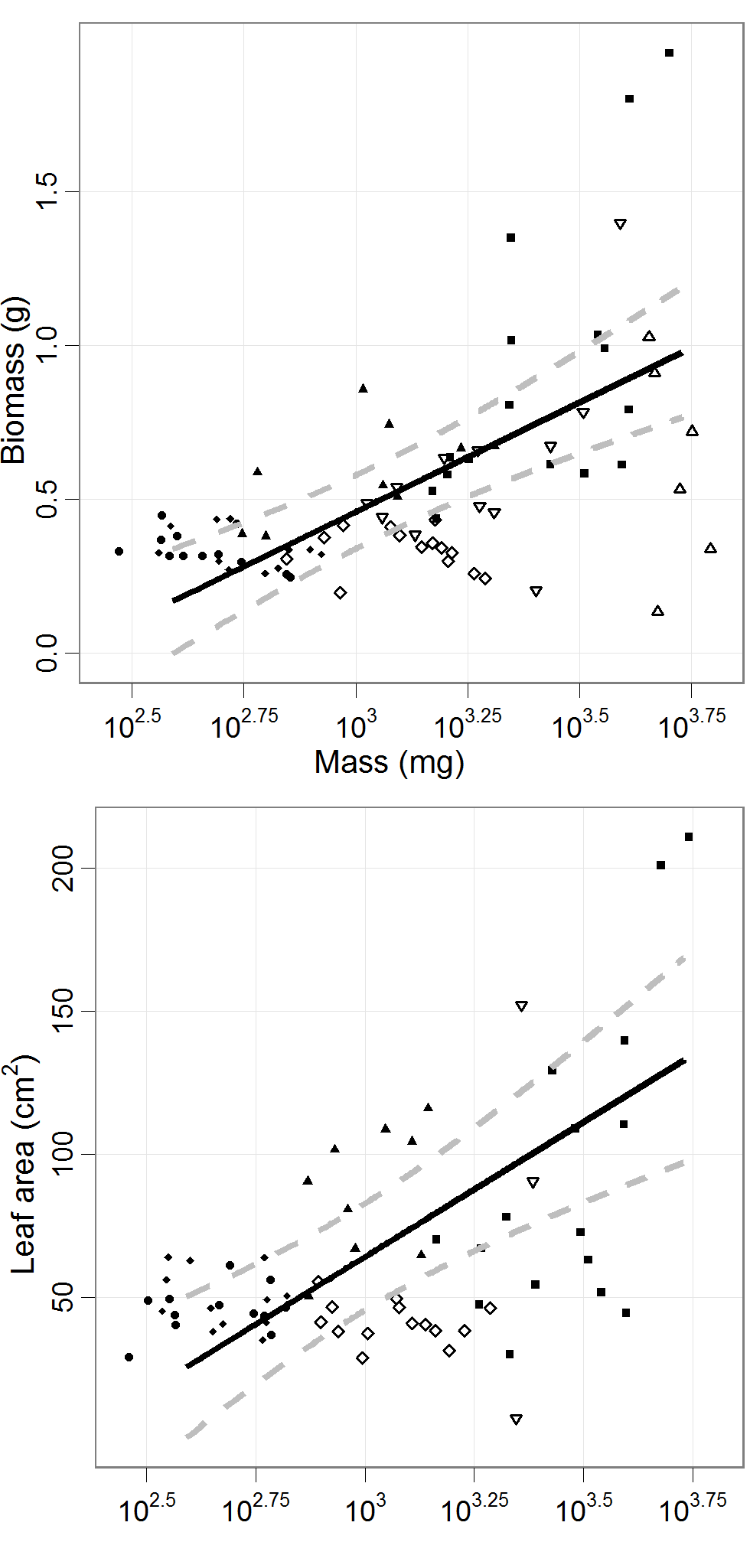

Supplement: Figure S6 — Relationship between growth and seed mass in daily watering without SM. The relationship of biomass and leaf area as a function of seed mass in the daily watering treatment (mean ±95% CI). Unequal variance was accounted for using a weighted variance for each species. Leaf area was based on the last harvest after 60 days. Points represent individual seedlings. (PT: upward triangle, DL: closed square, PM: downward triangle, HN: open diamond, SB: closed triangle, SP: closed diamond, SA: closed circle) (TIF) [file pone.0070287.s006.tif]

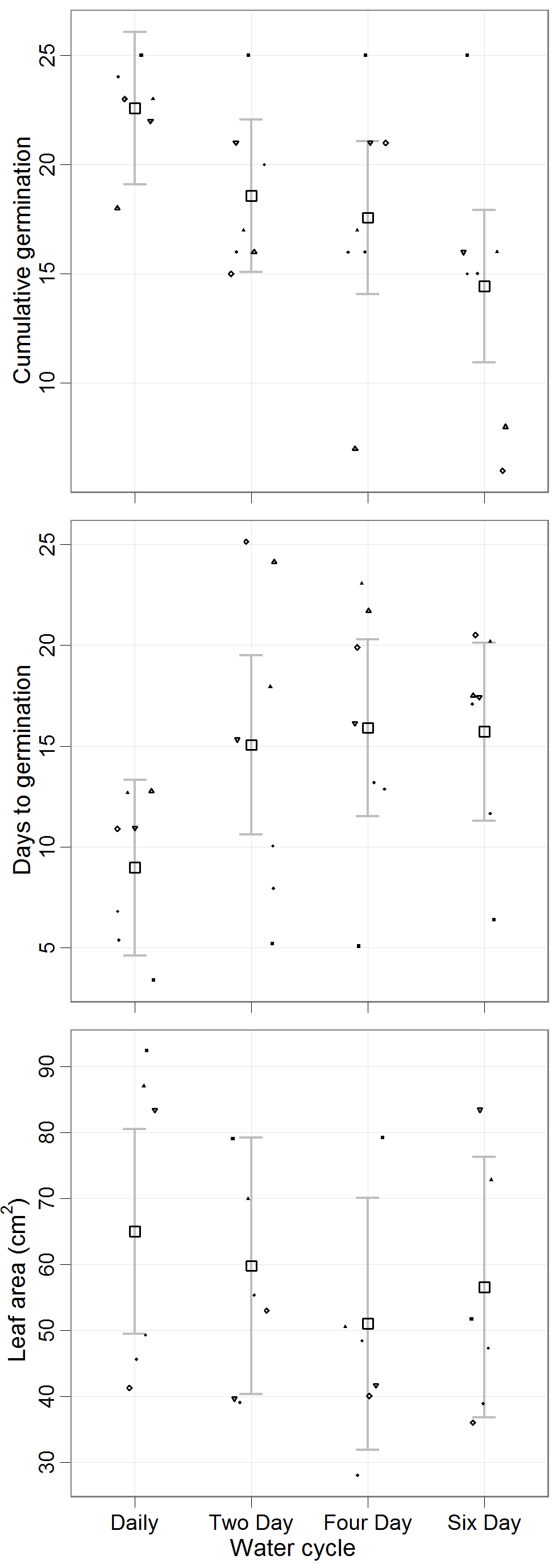

Supplement: Figure S7 — Effect of watering frequency on germination and growth without SM. Cumulative germination, days to germination and final seedling leaf area calculated in each treatment. Variation in species and treatments was accounted for with a random effect for species in treatment. Leaf area was calculated from the last harvest after 60 days. The large open squares represent the pooled mean (±95% CI) of the eight Bornean climax species. All three traits were negatively affected by infrequent watering. Grey points represent species means for each variable in each treatment. The points are jittered for readability. (PT: upward triangle, DL: closed square, PM: downward triangle, HN: open diamond, SB: closed triangle, SP: closed diamond, SA: closed circle) (TIF) [file pone.0070287.s007.tif]

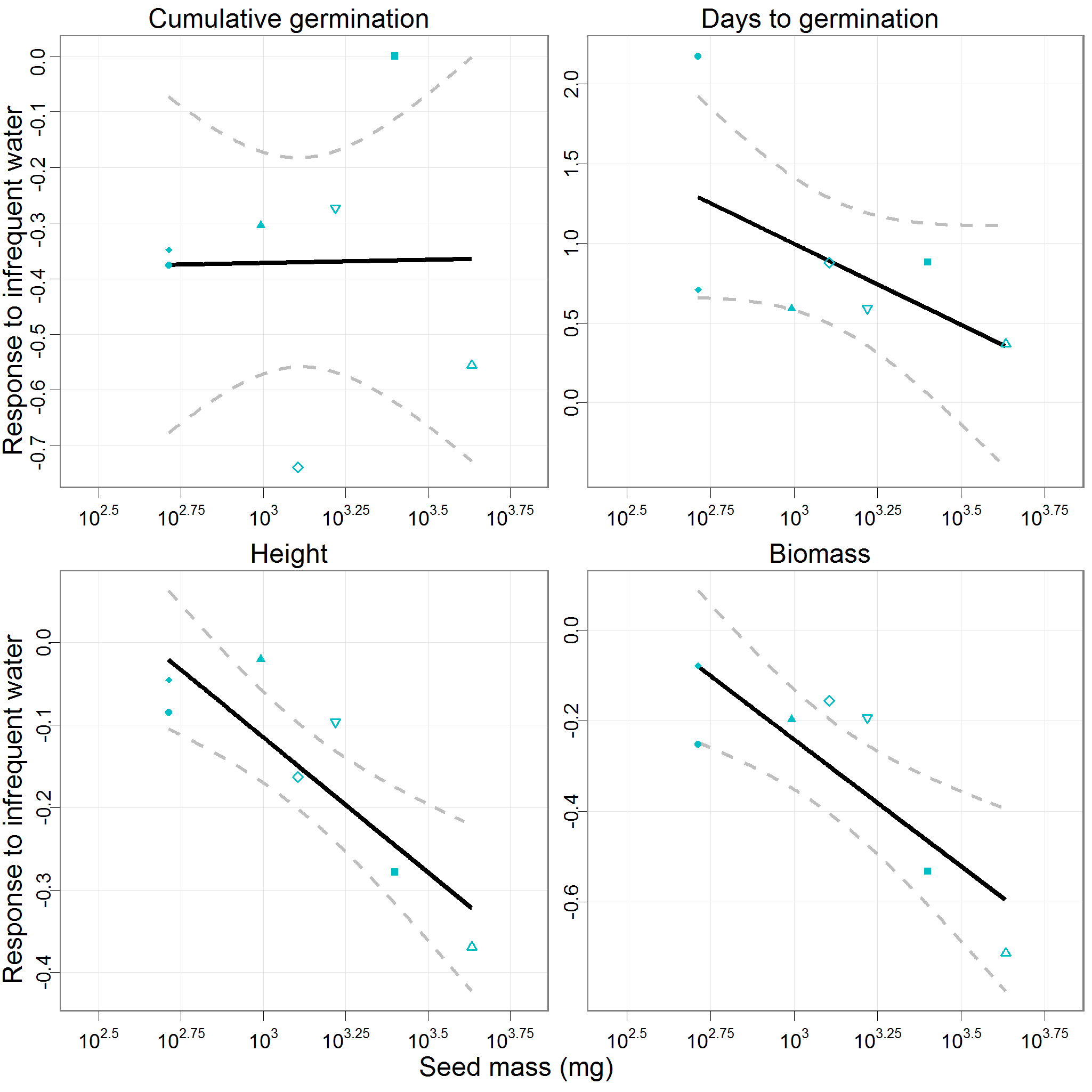

Supplement: Figure S8 — Relative response to infrequent water as a function of seed mass without SM. Response to infrequent watering (i.e. the relative difference between daily and six-day watering) as a function of seed mass for cumulative germination, days to germination and seedling height and biomass (mean ±95% CI). No relationship existed between cumulative germination and seed mass. Germination of large seeds was more resistant to infrequent watering, but after germination large-seeded species had significantly greater declines in growth due to infrequent watering. Points represent mean values for each species. (PT: upward triangle, DL: closed square, PM: downward triangle, HN: open diamond, SB: closed triangle, SP: closed diamond, SA: closed circle) (TIF) [file pone.0070287.s008.tif]

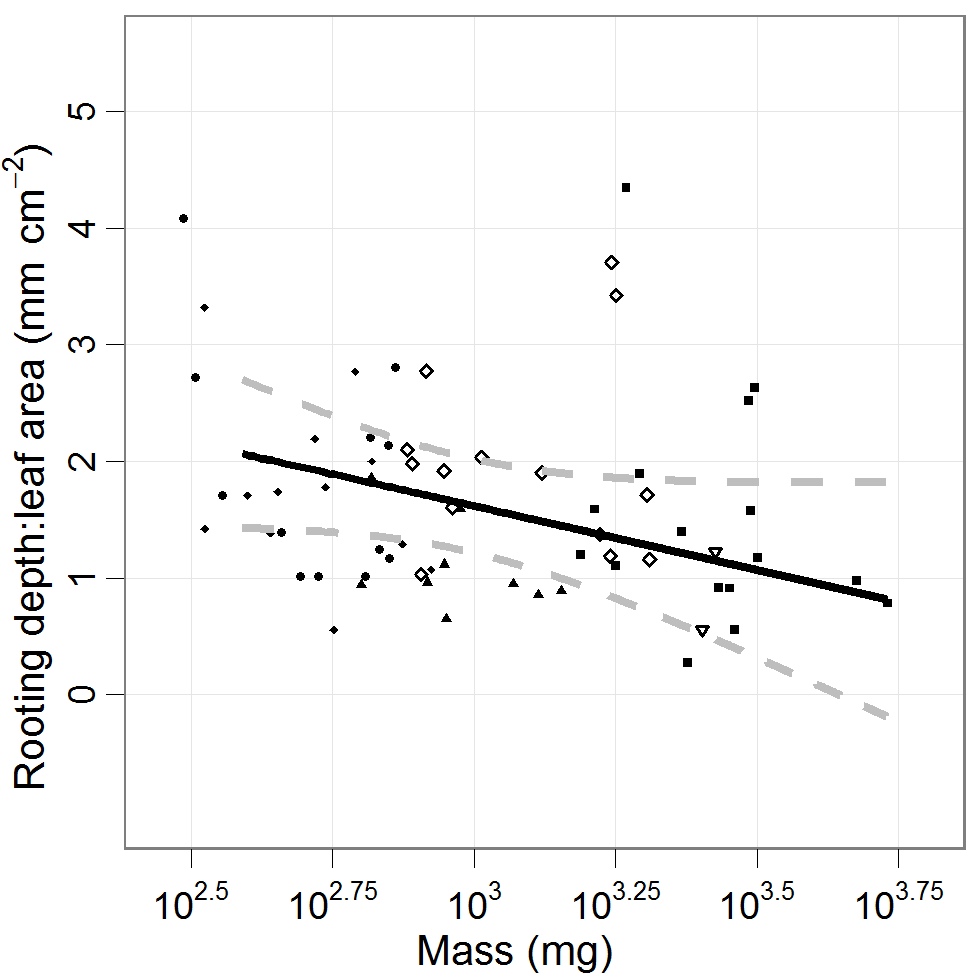

Supplement: Figure S9 — Rooting depth per leaf area as a function of seed mass without SM. Root depth per leaf area (mean ±95% CI) as a function of seed mass. ). Unequal variance was accounted for using a weighted variance for each species. Large-seeded species produce much larger leaves increasing transpiration and water demand without proportionally larger rooting depth, leaving them susceptible to breaks in hydraulic conductivity and water stress. Points represent individual seedlings. (PT: upward triangle, DL: closed square, PM: downward triangle, HN: open diamond, SB: closed triangle, SP: closed diamond, SA: closed circle) (TIF) [file pone.0070287.s009.tif]
