# Supplementary material for: The Influence of Variable Rainfall Frequency on Germination and Early Growth of Shade-Tolerant Dipterocarp Seedlings in Borneo
Source: PLoS One. 2013 Jul 24;8(7):e70287. doi: 10.1371/journal.pone.0070287 (PMC3722165; doi:10.1371/journal.pone.0070287)
Supplement: Table S1 — Summary of significance for parameters explaining soil matric potential. Species was removed from the model as it was never significant nor did it improve the fit of the model. (DOCX) [file pone.0070287.s010.docx]

| **Parameter** | **Num. DF** | **Denom. DF** | **F** | **P** |
| --- | --- | --- | --- | --- |
| Treat | 3 | 1231 | 33.66 | <0.0001 |
| Pre-watering | 1 | 1231 | 204.68 | <0.0001 |
| Day | 1 | 1231 | 110.84 | <0.0001 |
| Pre-watering*treat | 3 | 1231 | 11.84 | <0.0001 |
| Day*treat | 3 | 1231 | 7.03 | 0.0001 |
| Pre-watering*day | 1 | 1231 | 20.33 | <0.0001 |
| Pre-watering*day*treat | 3 | 1231 | 3.67 | 0.0120 |
